# ICP, PRx, CPP, and ∆CPPopt in pediatric traumatic brain injury: the combined effect of insult intensity and duration on outcome

**DOI:** 10.1007/s00381-023-05982-5

**Published:** 2023-06-03

**Authors:** Teodor Svedung Wettervik, Fartein Velle, Anders Hånell, Timothy Howells, Pelle Nilsson, Anders Lewén, Per Enblad

**Affiliations:** Department of Medical Sciences, Section of Neurosurgery, Uppsala University, SE-751 85 Uppsala, Sweden

**Keywords:** Cerebral autoregulation, Cerebral perfusion pressure, Intracranial pressure, Traumatic brain injury

## Abstract

**Purpose:**

The aim was to investigate the combined effect of insult intensity and duration, regarding intracranial pressure (ICP), pressure reactivity index (PRx), cerebral perfusion pressure (CPP), and optimal CPP (CPPopt), on clinical outcome in pediatric traumatic brain injury (TBI).

**Method:**

This observational study included 61 pediatric patients with severe TBI, treated at the Uppsala University Hospital, between 2007 and 2018, with at least 12 h of ICP data the first 10 days post-injury. ICP, PRx, CPP, and ∆CPPopt (actual CPP-CPPopt) insults were visualized as 2-dimensional plots to illustrate the combined effect of insult intensity and duration on neurological recovery.

**Results:**

This cohort was mostly adolescent pediatric TBI patients with a median age at 15 (interquartile range 12–16) years. For ICP, brief episodes (minutes) above 25 mmHg and slightly longer episodes (20 min) of ICP 20–25 mmHg correlated with unfavorable outcome. For PRx, brief episodes above 0.25 as well as slightly lower values (around 0) for longer periods of time (30 min) were associated with unfavorable outcome. For CPP, there was a transition from favorable to unfavorable outcome for CPP below 50 mmHg. There was no association between high CPP and outcome. For ∆CPPopt, there was a transition from favorable to unfavorable outcome when ∆CPPopt went below −10 mmHg. No association was found for positive ∆CPPopt values and outcome.

**Conclusions:**

This visualization method illustrated the combined effect of insult intensity and duration in relation to outcome in severe pediatric TBI, supporting previous notions to avoid high ICP and low CPP for longer episodes of time. In addition, higher PRx for longer episodes of time and CPP below CPPopt more than −10 mmHg were associated with worse outcome, indicating a potential role for autoregulatory-oriented management in pediatric TBI.

## Introduction

Traumatic brain injury (TBI) in children continues to cause a significant burden of mortality and morbidity [1]. Neurointensive care (NIC) aims to improve clinical outcome by avoiding secondary insults such as elevated intracranial pressure (ICP) and insufficient cerebral perfusion pressure (CPP) [2–4]. Current pediatric TBI guidelines suggest to keep ICP below 20 mmHg and CPP at least above 40 mmHg [2, 3]. However, the level of evidence is low, as these suggestions rely on a very limited number of observational studies [5–7]. In addition, one major consideration is the potential age-dependent effect of what constitutes an optimal or dangerous ICP and CPP [8, 9]. The range of normal arterial blood pressure (ABP) or CPP as well as the capacity and plateau level for cerebral pressure autoregulation vary extensively throughout childhood [10]. Although a “safe” lower CPP threshold may be anticipated on an epidemiological level for certain pediatric age groups in TBI, adequate cerebral blood flow (CBF) cannot be guaranteed [9, 10]. What defines a sufficient CBF in pediatric TBI in different age groups is not well-determined, since “normal” CBF is highly varied throughout childhood during the different phases of cerebral development and maturation [9, 10]. Consequently, the combined role of CPP and the cerebral pressure autoregulatory status has received increased interest as means to better optimize CBF in pediatric TBI [11]. Much of this interest and work stem from research on adult TBI. Particularly, emerging evidence supports the role of the pressure reactivity index (PRx) as a feasible and valid metric for cerebral pressure autoregulation [12–14]. PRx is defined as the correlation between ABP and ICP, where negative values indicate preserved pressure autoregulation and vice versa [12]. It has been demonstrated that PRx varies with CPP in a U-shaped fashion [13, 15–17], where the CPP with the lowest (best) PRx value has been considered optimal (CPPopt). In adult TBI, CPP close to CPPopt has been associated with better brain tissue oxygenation [18], energy metabolism [17], and outcome [13, 15, 16]. Recently, the feasibility study (COGiTATE) was published with promising results for CPPopt-oriented management [19]. In pediatric TBI, it has also been found that autoregulatory disturbances are common and correlate with worse cerebral physiology and outcome [11, 20–22]. In a few smaller exploratory pediatric TBI studies, higher PRx and greater distance between actual CPP and CPPopt were associated with increased mortality and unfavorable outcome [23–27]. More granular studies are needed to better determine the role of these autoregulatory variables in pediatric TBI. In this study, we aimed to better elucidate the combined effect of insult intensity and duration for the NIC variables ICP, PRx, CPP, and CPPopt on clinical outcome in pediatric TBI, by using a granular visualization method, originally developed by Guiza et al. [28] and later adapted by our group [14].

## Materials and methods

### Patients and study design

Pediatric patients (age < 18 years) with severe TBI (unconscious post-resuscitation (Glasgow Coma Scale ≤ 8) or later after deterioration) who were admitted between 2007 and 2018 to the Department of Neurosurgery, Uppsala University Hospital, Sweden, and received ICP monitoring with at least 12 cumulative hours of ICP data during the first 10 days, were eligible for this observational study. Out of 78 pediatric TBI patients below 18 years of age, 64 patients had received an ICP monitor, but 3 of these were excluded because of unavailable or insufficient ICP data. Hence, the final study population included 61 pediatric TBI patients.

### Management protocol

The ICP-/CPP-oriented management protocol for pediatric TBI in Uppsala University Hospital has been described in detail in previous studies [29, 30]. Treatment goals were ICP ≤ 20 mmHg, pO_2_ > 12 kPa, arterial glucose 5–10 mmol/L, electrolytes within normal ranges, and body temperature < 38 °C. CPP was targeted at or above 60 mmHg in older children, but lower thresholds down to 45–50 mmHg were allowed in small children. Severe (unconscious) pediatric TBI patients were intubated and mechanically ventilated and received ICP and arterial blood pressure (ABP) monitoring. An external ventricular drainage (EVD) was chosen in first place for ICP monitoring, provided that the ventricles were not too small. The EVD was kept closed or intermittently opened if needed the first days when the risk of expanding hematomas was highest. Later, the EVD was kept open and cerebrospinal fluid was drained continuously at a pressure level of 15 mmHg. In case of compressed ventricles initially, a parenchymatous pressure device was used instead. In those cases, an EVD was sometimes inserted later for drainage if ICP was high. Intracranial hemorrhagic lesions with mass effect were surgically evacuated. If ICP was refractory high, thiopental infusion and decompressive craniectomy (DC) were last-tier treatments.

### Data acquisition

ICP was monitored with either an EVD (HanniSet, Xtrans, Smith Medical GmbH, Grasbrunn, Germany) or with an intraparenchymal sensor device (Codman ICP Micro-Sensor, Codman & Shurtleff, Raynham, MA). In cases when both an EVD and an intraparenchymal monitor were in place, ICP was analyzed from the EVD. ABP was measured in the radial or femoral artery at heart level. Pressure reactivity index was calculated as the 5-min correlation of 10-s averages of ICP and MAP [12, 13]. CPPopt was continuously calculated as the CPP with the lowest PRx the last 4 h [15, 16]. ∆CPPopt (the difference between actual CPP and CPPopt) was continuously calculated for every minute of available CPPopt data. CPPopt could be calculated during 53% of the total monitoring time with available CPP data, similar to previous studies [13, 14, 31]. Monitoring data from the first 10 days post-injury, but not later, were included. The physiological data were collected at 100 Hz in the Odin software [32]. The physiological data were automatically and manually cleaned from artefacts.

### Outcome

Clinical outcome was assessed 6 months post-injury, using the Glasgow Outcome Scale (GOS), containing five categories of global outcome, from death (1) to good recovery (5) [33, 34]. Favorable/unfavorable outcome was defined as GOS 4–5/1–3. The assessment was done by specially trained personnel with structured telephone interviews with the patient or their next-of-kin.

### Visualization method

The relationship between insult intensity and duration with outcome was visualized using the method originally described by Guiza and colleagues [28, 35], which was implemented and adapted using custom written R-scripts [14]. The plots were based on ICP/PRx/CPP/CPPopt data during the first 10 days post-injury. An insult was defined as data below/above the threshold for CPP and ∆CPPopt and above the threshold for ICP and PRx. Scanning the dataset resulted in a set of insults with the intensity determined by the threshold used when they were detected and the duration by how long they remained above or below this threshold. For each combination of intensity and duration, the correlation with outcome was then determined as previously described [28, 35]. Gaussian smoothing with a standard deviation of 1 was applied to the matrix of correlation values to reduce high-frequency noise. The final correlation values were visualized using the jet color scale, while regions with insufficient data (less than 20 patients for a given combination of threshold and duration) were colored white. Pixels associated with a negative correlation coefficient, i.e., when more episodes of the specific combination of exceeding that intensity for a certain duration were associated with unfavorable outcome, were considered an insult.

### Statistical analyses

Nominal variables were presented as numbers (proportions) and ordinal/continuous variables as medians (interquartile range (IQR) or range). The visualization plots were descriptive for the relation between insult intensity and duration vs. GOS, but did not allow for more formal significance testing. The limited cohort size of pediatric TBI patients inhibited us from proceeding with multiple logistic analyses, which was previously done in studies with adults [14]. There were only a few missing observations (5 patients without available CPPopt), which were excluded from the analyses. The statistical analyses were performed in SPSS version 28 (IBM Corp, Armonk, NY, USA).

## Results

### Patients, admission variables, treatments, and clinical outcome

As demonstrated in Table 1, median age was 15 (IQR 12–16/range 0.5–17) years and the majority (*n* = 40 (66%)) were male. Median Glasgow Coma Scale was 7 (IQR 6–8) at admission and Rotterdam score was in median 4 (IQR 3–4). The majority (*n* = 35 (57%) received a Codman ICP monitor and the remaining patients an EVD (*n* = 16 (26%)) or both (*n* = 10 (16%)). All patients were ICP-monitored for more than 24 h and the median number of days with monitoring was 10 (IQR 6–14). Eighteen (30%) patients were treated with thiopental infusion and 11 (18%) were operated with (DC). After 6 months, 2 (3%) were deceased, 52 (85%) had recovered favorably, and GOS was in median 5 (IQR 4–5).

**Table 1:** Demographics, admission variables, treatments, and clinical outcome

**Variables**	**Patients**
Patients, *n*	61
Age, median (IQR/range)	15 (12–16/0.5–17)
Sex–male, *n* (%)	40 (66%)
GCS, median (IQR)	7 (6–8)
Pupillary status (normal/one unreactive/both unreactive), *n* (%)	50/2/9 (82/3/15%)
Rotterdam score, median (IQR)	4 (3–4)
Codman/EVD/both, *n* (%)	35/16/10 (57/26/16%)
ICP monitoring (days), median (IQR)	10 (6–14)
Thiopental (yes), *n* (%)	18 (30%)
DC (yes), *n* (%)	11 (18%)
GOS, median (IQR)	5 (4–5)
Favorable outcome, *n* (%)	52 (85%)
Mortality, *n* (%)	2 (3%)

*DC* decompressive craniectomy, *GCS* Glasgow Coma Scale, *EVD* external ventricular drainage, *GOS* Glasgow Outcome Scale, *ICP* intracranial pressure, *IQR* interquartile range, *TBI* traumatic brain injury

### ICP, PRx, CPP, and ∆CPPopt–the role of insult intensity and duration in relation to clinical outcome

**Fig. 1:**
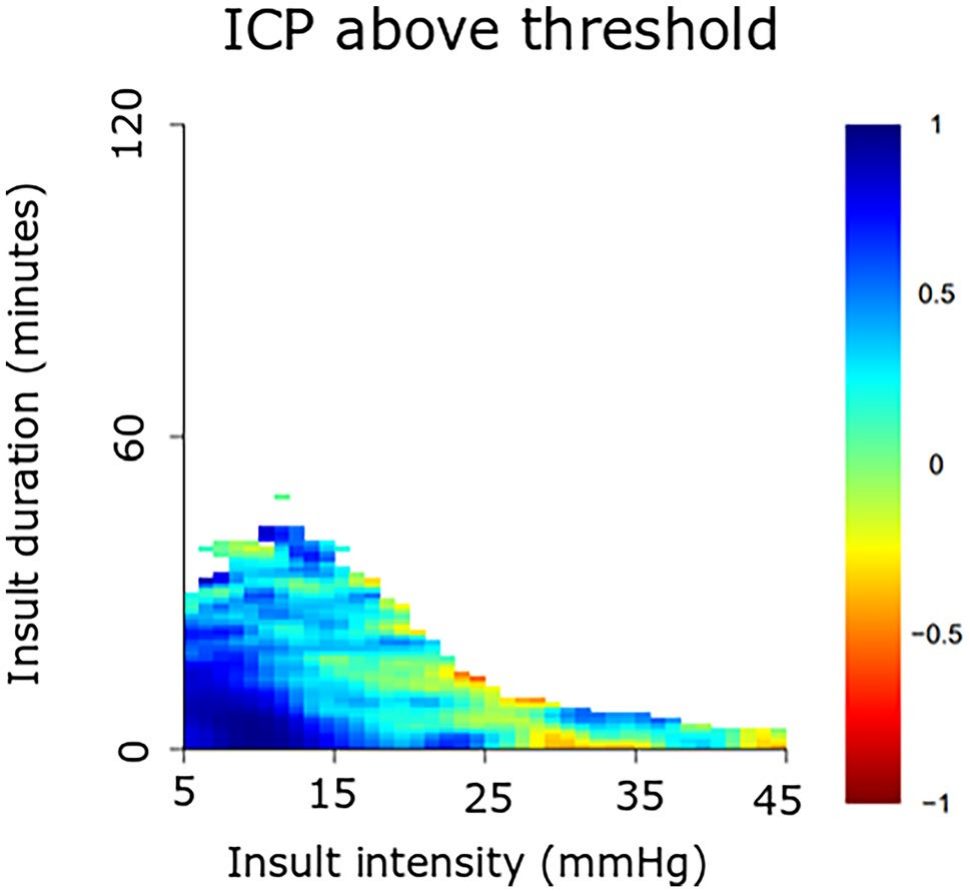
ICP insults in relation to clinical outcome in pediatric TBI. The plot illustrates the association between ICP insult intensity and duration with clinical outcome (GOS). The jet color scale on the right indicates the correlation between the combination of intensity and duration with clinical outcome (GOS). Blue represents favorable and red color unfavorable outcome. Regions with insufficient data were colored white. GOS, Glasgow Outcome Scale. ICP, intracranial pressure. TBI, traumatic brain injury

**Fig. 2:**
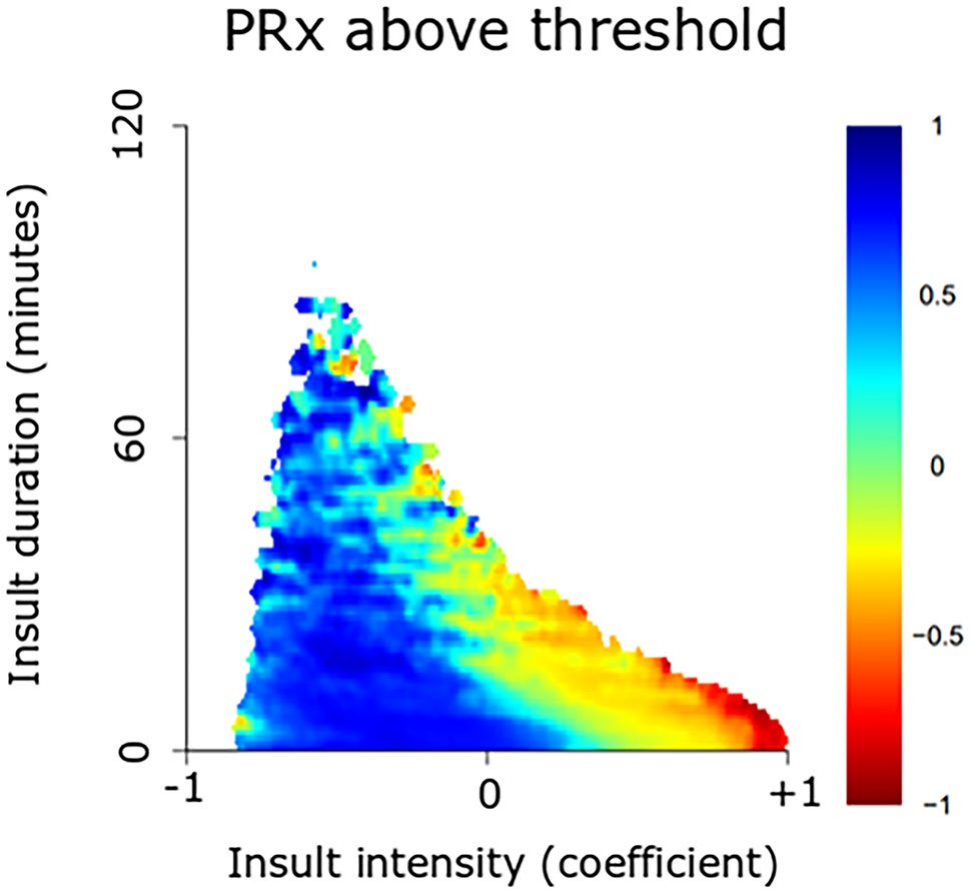
PRx insults in relation to clinical outcome in pediatric TBI. The plot illustrates the association between PRx insult intensity and duration with clinical outcome (GOS). The jet color scale on the right indicates the correlation between the combination of intensity and duration with clinical outcome (GOS). Blue represents favorable and red color unfavorable outcome. Regions with insufficient data were colored white. GOS, Glasgow Outcome Scale. PRx, pressure reactivity index. TBI, traumatic brain injury

**Fig. 3:**
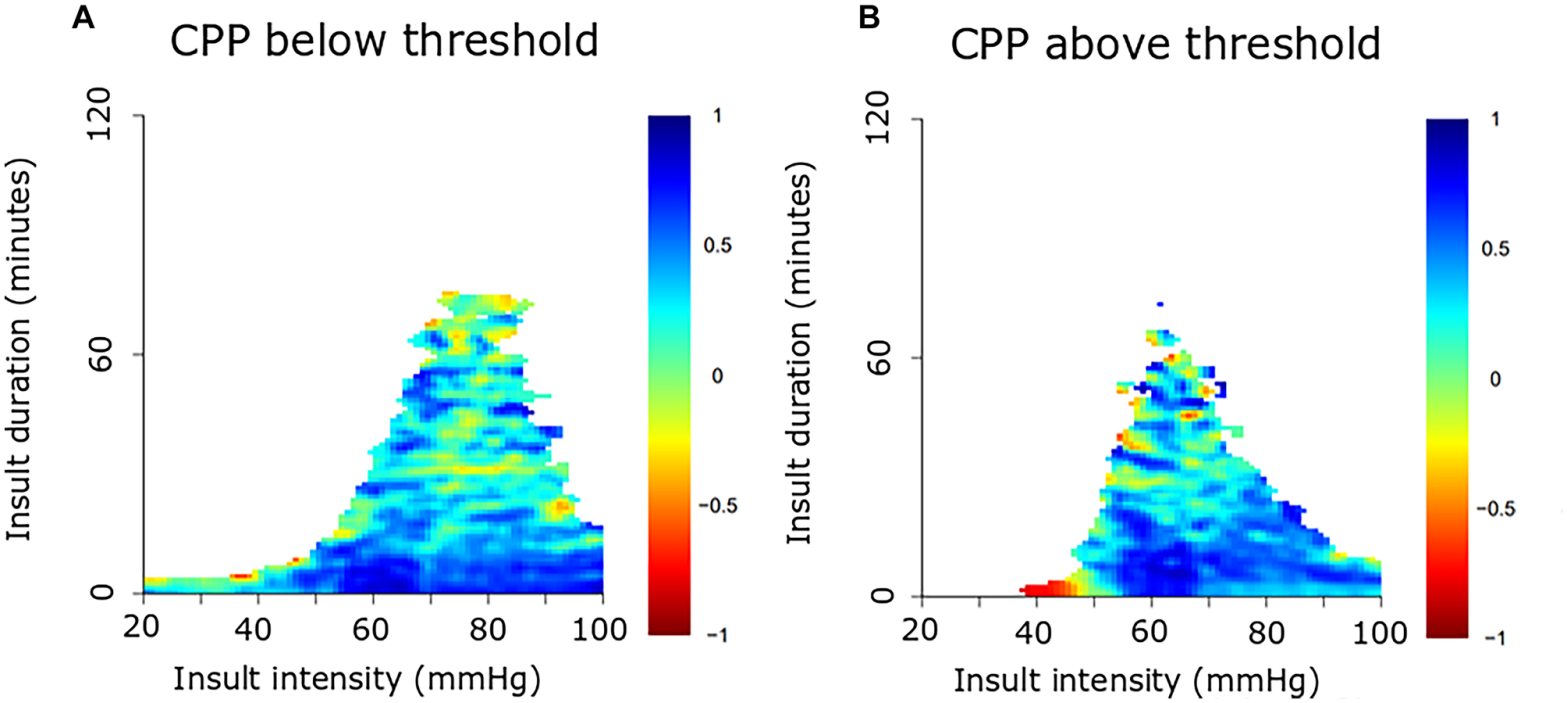
CPP insults in relation to clinical outcome in pediatric TBI. The plot illustrates the association between CPP (**A**, **B**) insult intensity and duration with clinical outcome (GOS). The jet color scale on the right indicates the correlation between the combination of intensity and duration with clinical outcome (GOS). Blue represents favorable and red color unfavorable outcome. Regions with insufficient data were colored white. CPP, cerebral perfusion pressure. CPPopt, optimal CPP. ∆CPPopt, CPP-CPPopt. GOS, Glasgow Outcome Scale. TBI, traumatic brain injury

**Fig. 4:**
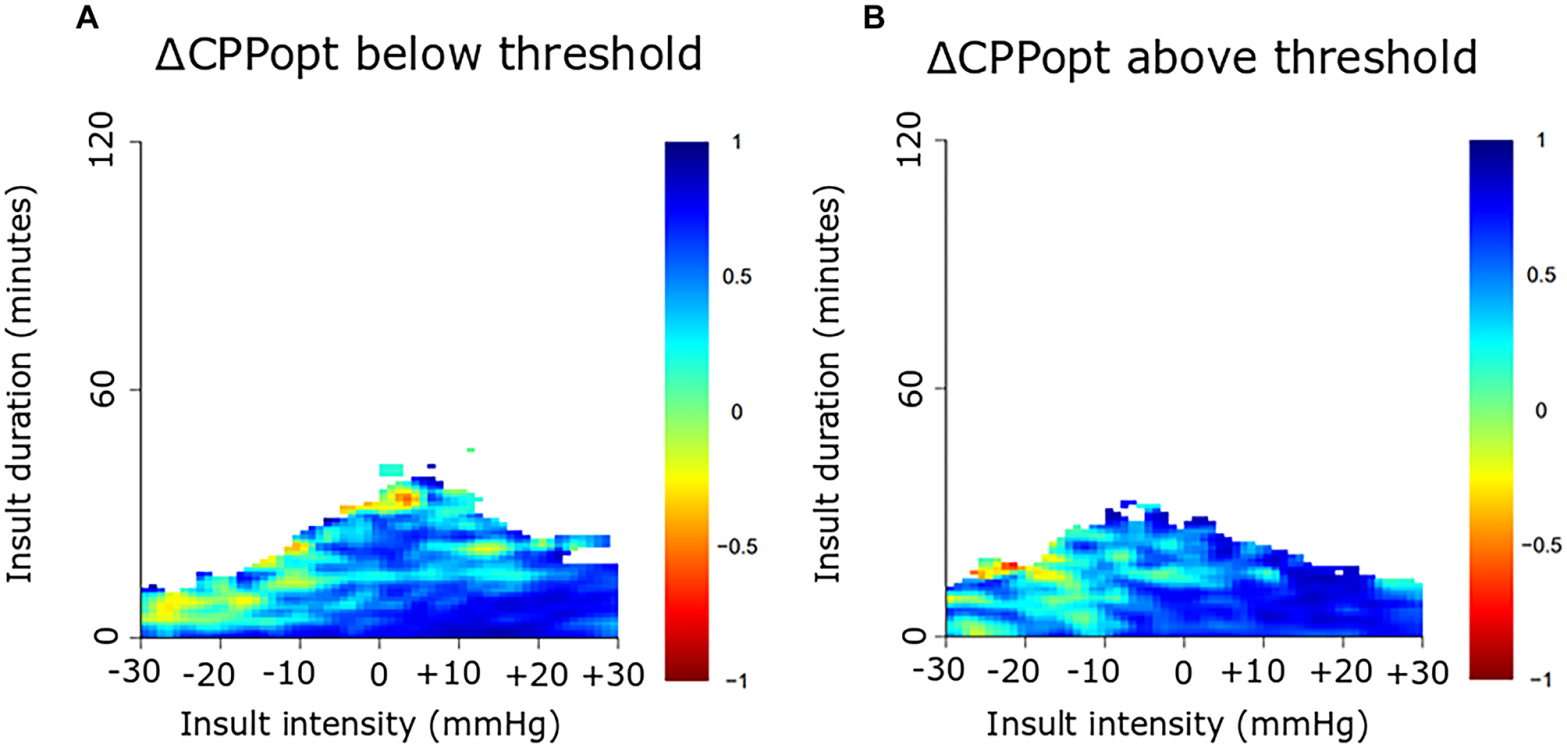
∆CPPopt insults in relation to clinical outcome in pediatric TBI. The plot illustrates the association between ∆CPPopt (**A**, **B**) insult intensity and duration with clinical outcome (GOS). The jet color scale on the right indicates the correlation between the combination of intensity and duration with clinical outcome (GOS). Blue represents favorable and red color unfavorable outcome. Regions with insufficient data were colored white. CPP, cerebral perfusion pressure. CPPopt, optimal CPP. ∆CPPopt, CPP-CPPopt. GOS, Glasgow Outcome Scale. TBI, traumatic brain injury

The insult intensity/duration plots are demonstrated for ICP (Fig. 1), PRx (Fig. 2), CPP (Fig. 3A–B), and ∆CPPopt (Fig. 4A–B). For ICP (Fig. 1), brief episodes (minutes) above 25 mmHg were associated with unfavorable outcome. Similar was true for slightly lower ICP (20–25 mmHg) for longer insult durations (20 min). For PRx (Fig. 2), there was a transition from favorable to unfavorable outcome for short episodes (minutes) of PRx above approximately 0.25, whereas slightly lower values were also associated with unfavorable outcome if they persisted for a longer period of time (30 min). For CPP (Fig. 3A–B), there was a transition from favorable to unfavorable outcome for CPP below 40–50 mmHg, although there were very few insults for such low values. There was no association between high CPP and worse outcome (Fig. 3B). For ∆CPPopt (Fig. 4A), there was a transition from favorable to unfavorable outcome when ∆CPPopt went below −10 mmHg. No association was found for positive ∆CPPopt values and worse outcome (Fig. 4B).

## Discussion

In this study, on pediatric (particularly adolescent) patients with severe TBI, we explored the combined role of insult intensity and duration for ICP, PRx, CPP, and CPPopt in relation to clinical outcome. Of particular interest, the results clearly illustrated the transition from favorable to unfavorable outcome with longer episodes of elevated PRx. There was also a trend towards unfavorable outcome when ICP went above 20–25 mmHg and CPP below 40–50 mmHg, consistent with the pediatric BTF guidelines [3]. In addition, there was a clear-cut transition towards unfavorable outcome when ∆CPPopt went below −10 mmHg, which holds promise for CPPopt as a potential autoregulatory-oriented target in pediatric TBI.

Regarding ICP, there was a transition towards unfavorable outcome when ICP exceeded 20–25 mmHg for longer durations (20 min), whereas higher intensities such as 25 mmHg were associated with unfavorable outcome even for very brief episodes (minutes). The transition thresholds are consistent to those found by Guiza et al. who conducted a similar insult intensity/duration analysis of ICP in a multi-center pediatric TBI cohort (*n* = 99) [28]. Furthermore, it appeared both in the current study and the one by Guiza [28] that the transition towards unfavorable outcome for brief (minutes) ICP insults occurred at a slightly lower ICP around 15–20 mmHg in the pediatric population, whereas the study by Guiza and a previous one from our group have indicated that this transition starts at a higher ICP closer to 20–25 mmHg in adults [14, 28]. It is possible that children have a lower tolerance for ICP elevation than adults [9].

Only a handful of studies have investigated PRx [21, 23, 24, 26, 27] or the low-frequency variant (LAx) [36] in pediatric TBI. This is the first study to analyze the combined effect of insult intensity/duration of PRx in relation to outcome in pediatric TBI, although Flechet conducted a similar analysis using the low-resolution metric LAx [36]. Interestingly, and consistent with the LAx analyses [36], our visualization plot very clearly illustrates the gradual transition towards unfavorable outcome with higher PRx for longer episodes of time. This highlights the importance of a preserved pressure autoregulation for neurological recovery. In addition, the transition towards unfavorable outcome began with brief episodes (minutes) of PRx above 0.25, whereas this transition started at a lower PRx around zero in an adult cohort study by our group [14]. It is possible that pediatric TBI patients are more resilient and tolerate autoregulatory disturbances better than adult patients. It also suggests that PRx should routinely be monitored and possibly optimized during NIC [15, 17, 18, 37, 38].

Regarding CPP, although there were only a few episodes of CPP below 40 to 50 mmHg, a trend towards unfavorable outcome was found for such insults in our study, consistent with the pediatric TBI guidelines [3]. Interestingly, unlike adult TBI patients where CPP between 60 and 70 mmHg appears most favorable and both higher and lower values are associated with worse outcome on a group level [14, 39], high CPP did not appear dangerous in our pediatric TBI cohort. In more granular adult TBI studies, it has been shown that high CPP is favorable in patients with intact pressure autoregulation [32], possibly since these patients are able to protect themselves from hyperemia via vasoconstriction. On the contrary, those with lost pressure autoregulation do better with slightly lower CPP [32], possibly as higher CPP provokes hyperemia and edema formation. It is conceivable that our cohort of pediatric TBI patients exhibited healthier and more pressure active vessels on a general level, which would explain why high CPP did not appear dangerous on a group level. Our findings were consistent with the recent study by Smith et al. on severe TBI in pediatric patients [21], which demonstrated that PRx was primarily high/disturbed for low CPP, but unlike the situation with a U-shaped curve in adult TBI, PRx did not increase much with higher CPP values. Thus, high CPP may be less dangerous in pediatric TBI, most likely because pressure autoregulation is better preserved for higher CPP. Furthermore, there was a transition towards unfavorable outcome for ∆CPPopt below −10 mmHg that by visual inspection appeared more clear-cut and robust than the CPP plots. In addition, analysis of PRx and CPPopt in the Uppsala pediatric TBI cohort in a more traditional way showed similar results as in the current study [22].

Considering the great age range in our cohort and that both the regulation and the absolute requirement of CBF are highly variable throughout childhood [10], it is not surprising that an autoregulatory-derived target could yield a better and more individualized surrogate measure of CBF than fixed CPP. However, CPPopt could only be calculated during 53% of the monitoring time with ICP/CPP, which currently limits its validity, although certain modelling methods have been attempted to address this [40]. Further multimodality monitoring studies of CPPopt targets in relation to brain tissue oxygenation and microdialysis as well as prospective clinical trials using CPPopt are needed to further explore this potential treatment target in pediatric TBI.

### Methodological considerations

There were several strengths with this study. First, it was based on a relatively large patient cohort with high-frequency monitoring data, as compared to previous pediatric studies on PRx and CPPopt [23–27]. Second, this was also the first study to evaluate the combined role of insult intensity and duration for PRx and ∆CPPopt in pediatric TBI, which allowed for a more in-depth investigation for these variables.

There were also some limitations. First, patient heterogeneity is of great concern, both considering the great anatomical and physiological developmental changes that occur throughout the entire childhood and the heterogeneity of TBI itself as a disease. The study was also based on a single-center cohort, which limits the external validity. Second, the visualization plots provided robust visual guidance, but did not allow for significance testing. Third, our analyses between physiological insults and outcome were associations, which could partly reflect causality, but also the extent of underlying brain injury, current management, and the treatment thresholds used at our NIC unit. This was particularly a limiting factor, since we were unable to proceed with multiple regression analyses to evaluate the independent association of the amount of insult intensity/duration on outcome. Fourth, the visualization plots were to some extent sensitive to noise. This was particularly evident in the PRx and ∆CPPopt plots, since both variables are associated with a low signal-to-noise ratio. CPPopt could also only be calculated for 53% of the CPP monitoring time, which reduced the possibility to analyze longer insult durations. In addition, longer and more extreme intensity insults were also less frequent, which reduced the reliability of the corresponding areas in the plots to some extent. Fifth, there has been a suspicion that DC could influence autoregulatory measures but previous studies indicate that DC should not reduce the validity of PRx and consequently CPPopt [41] and we therefore chose not to exclude DC patients from our analyses. Similar concerns have been raised in case of an open EVD, but preliminary studies suggest that opening an EVD does not reduce the reliability of PRx and thus CPPopt [42]. Lastly, it might have been better to use the GOS-E for pediatrics [43], but our intention was not to present and analyze detailed clinical outcome results and instead we used the ordinary adult GOS adapted for children.

## Conclusions

This visualization method utilized in this study illustrates the combined effect of insult intensity and duration in relation to outcome in pediatric TBI. As expected, longer episodes of ICP above 20–25 mmHg and CPP below 40–50 mmHg were associated with unfavorable outcome. In addition, the importance of higher PRx for longer episodes of time and CPP more than 10 mmHg below CPPopt was clearly visualized and strongly associated with worse outcome, indicating a role for autoregulatory-oriented management in pediatric TBI.

## Data Availability

Data are not available due to legal restrictions.
